# Next generation sequencing based pathogen analysis in a patient with neurocysticercosis: a case report

**DOI:** 10.1186/s12879-018-3015-y

**Published:** 2018-03-06

**Authors:** Ping Liu, Xing Weng, Jiajia Zhou, Xiaolin Xu, Fangping He, Yue Du, Honglong Wu, Yanping Gong, Guoping Peng

**Affiliations:** 1 Department of Neurology, First Affiliated Hospital, Zhejiang University School of Medicine, 79 Qingchun Road, Hangzhou, 310003 China; 2 BGI-Shenzhen, Main Building, Bei Shan Industrial Zone, Yantian District, Shenzhen, Guangdong 518083 China; 3 BGI-Shanghai, 8th Floor, 26th Building, 3399 Lane, Kangxin Road, Pudong District, Shanghai, 200120 China; 4 Binhai Genomics Institute, Tianjin Translational Genomics Center, BGI-Tianjin, Tianjin, 300308 China

**Keywords:** Neurocysticercosis, Next generation sequencing, *Taenia solium*, Headache, Sensory stroke

## Abstract

**Background:**

Accurate and early diagnosis of neurocysticercosis (NCC) remains a challenge due to the heterogeneity of its clinical, immunological and imaging characteristics. The presence of cysticercus DNA in cerebrospinal fluid (CSF) of NCC patients has been previously detected via conventional PCR assays. To the best of our knowledge, the use of CSF Next-Generation Sequencing (NGS) based pathogen analysis in patients with NCC infection has never been reported.

**Case presentation:**

This study reports the clinical, imaging, and immunological features of a patient initially presenting with several months of headache who further developed a pure sensory stroke. NGS was used to detect the pathogen, and her CSF demonstrated the presence of *Taenia solium*-DNA. This finding was confirmed by a positive reaction to CSF cysticercosis antibodies. After antiparasitic treatment, secondary CSF NGS revealed the DNA index have dropped considerably compared to the initial NGS readings.

**Conclusions:**

NGS is a promising tool for the early and accurate diagnosis of central nervous system (CNS) infection, especially in the setting of atypical clinical manifestations. Further studies are required to evaluate the persistence of DNA in the CSF of patients.

## Background

Neurocysticercosis (NCC) is caused by infection of the human central nervous system (CNS) with the larval stage of *Taenia solium* [1]. The clinical manifestations of NCC are nonspecific and vary depending on the number and topography of lesions, as well as the host immune responses [2]. Ischemic cerebrovascular disease is a relatively common but under-recognised complication of NCC [3]. While accurate and early diagnosis of NCC remains challenging, Next-Generation Sequencing (NGS) offers a rapid, cost-effective and high-throughput DNA sequencing of pathogens, advancing both accurate diagnostic capabilities of clinicians and prospects of personalized medicine [4]. Here, we report one patient presenting with an atypical headache who proceeded to develop an ischemic stroke and was revealed to possess *T. solium* DNA in cerebrospinal fluid (CSF) via NGS.

## Case presentation

A 44-year-old female factory worker presented to our clinic complaining of a 1 year history of repeated, temporal and parietal headache characterized by bursting sensations. She had previously presented to a local hospital 6 months prior, in April 2016. The patient’s initial brain magnetic resonance imaging (MRI) and magnetic resonance angiography (MRA) were normal. CSF studies revealed 68 white cells per microliter and a pressure of 310 mmH_2_O. Viral encephalitis was suspected and she received acyclovir intravenously (500 mg Q8H for 7 days), but symptoms showed no signs of relief.

**Fig. 1:**
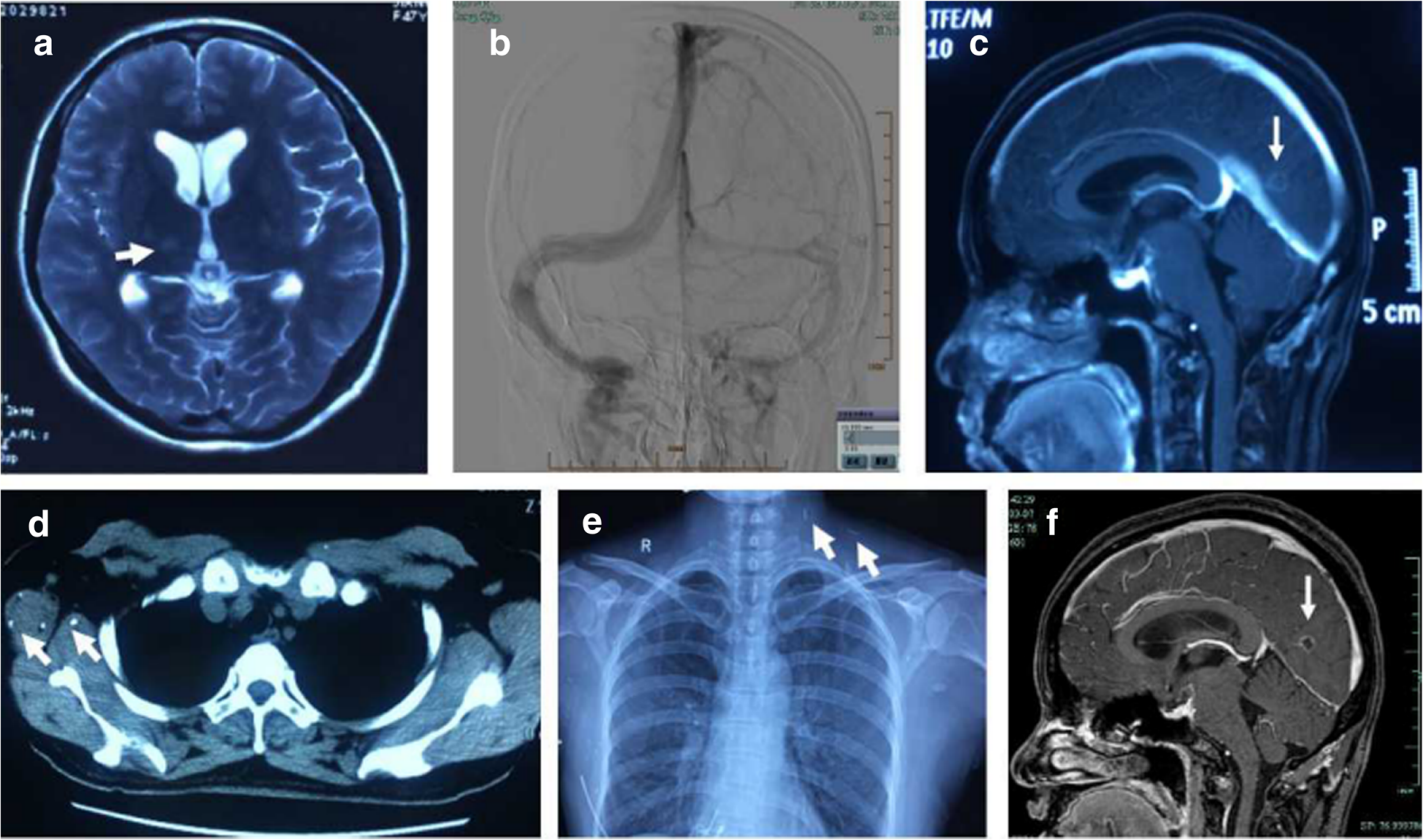
**a** The T2-weighted image showed a high signal of right thalamus. **b** DSA showed the probable underdevelopment on right lateral sinus while the venous system was smooth. **c** Cerebral MRI with contrast showed several small hyperintense lesions involving right cerebellar hemisphere and bilateral occipital, with ring enhancement of gadolinium. **d** The pulmonary CT showed multiple densities in the chest wall soft tissue. **e** Chest radiographs showed multiple calcification on the left side of the neck and right soft tissue of armpit chest wall. **f** The secondary MRI showed the nearly same small lesions with ring enhancement in the brain

The patient visited the local hospital again 3 months later, in July 2016, complaining of an aggravated headache and paroxysmal numbness of her left face and arm. She was diagnosed with an ischemic stroke of the right thalamus and administered oral aspirin 100 mg and atorvastatin calcium 20 mg daily (Fig. 1a). Her numbness improved while the headache showed no improvement.

She visited the local hospital for the third time 5 days prior to presenting to our hospital due to the severity of her headache on October 26, 2016. Lumbar puncture was remarkable for a CSF pressure greater than 400 mmH_2_O, white cell count of 40 per microliter, and a protein level of 1171 mg/L. Cerebral magnetic resonance venography (MRV) revealed underdevelopment of her right lateral sinus and sinus thrombosis was considered as a possible etiology. She was treated with low molecular heparin and her headache was slightly relieved. She was then admitted to our hospital for further treatment.

The patient had no significant past medical history, denied a history of blood transfusions and consumption of raw food. Her physical examination was unremarkable, and no abnormal neurologic signs apart from horizontal nystagmus was noted.

Laboratory tests revealed the proportion of eosinophils to be slightly elevated (3.35%) while other indices were within normal limits. HIV, syphilis, and Herpes simplex virus-1 and 2 antibodies were absent. In addition, the T-Cell spot test, purified protein derivative test for *Mycobacillus Tuberclosis* and Latex agglutination test for the cryptococcal capsular antigen were all negative. She underwent three lumbar punctures after admission to our clinic and results revealed a high intracranial pressure and an increased white cell count (Table 1).

**Table 1:** The results of 3 times lumbar puncture of the patient after admission in our hospital

Date	Pressure (mmH_2_O)	Nucleated cells (count/μl)	Neutrophilic granulocyte(%)	Lymphocyte(%)	Protein (g/L)	Glucose (mmol/l)	Chloride (mmol/l)
2016.11.3	330 ↑	100 ↑	10%	90%	0.649↑	2.2↓	124
2016.11.9	220 ↑	60 ↑	22%	78%	0.484↑	2.2↓	123
2016.11.14	265 ↑	50 ↑	45%	55%	0.482↑	2.3↓	125

Normal range: nucleated cells, 0~ 5/μl; protein, 0.15~ 0.45 g/L; glucose, 2.5~ 4.5 mmol/l; chloride, 120~ 131 mmol/l

No abnormalities were found on cerebral MRA, while MRV imaging suggested underdevelopment of the right lateral sinus. Digital subtraction angiography (DSA) revealed the arterial system to be normal and confirmed dysplasia of the right lateral sinus (Fig. 1b). Cerebral MRI with contrast revealed several small, hyperintense lesions involving the right cerebellar hemisphere and bilateral occipital lobe, with gadolinium ring enhancement (Fig. 1c). NGS of CSF sample was further carried out and suggested several possible pathogens including parasites and virus (Table 2). Serum was subsequently evaluated for presence of cysticercal antibodies and CSF also confirmed positivity, but negative for *Echinococcus granulosus*. Review of the patient’s previous imaging records revealed several additional anomalies: her pulmonary CT revealed multiple densities in chest wall soft tissue (Fig. 1d), and her chest radiographs take 6 months prior to admission to our hospital revealed multiple calcifications on the left side of the neck and right chest wall (Fig. 1e). A diagnosis of cerebral cysticercosis was therefore finally established. Oral praziquantel (2.5 mg/Kg/d × 7 days for one procedure) was administered thrice. Her headache was recovered markedly. Three months after she was discharged, her secondary cranial MR scan revealed persistence of the aforementioned ring-enhancing lesions (Fig. 1f). She underwent a repeat lumber puncture that revealed a CSF pressure of 220 mmH_2_O and 10 white blood cells per microliter. Meanwhile, CSF cysticercal antibodies remained positive. Repeat CSF NGS revealed that the *T. solium* index dropped considerably compared to initial NGS readings. On follow-up 6 months later, she reported no persisting symptoms.

**Table 2:** The results of NGS of CSF before and after praziquantel therapy

Type	Name	Coverage %		Depth		Number of unique reads	
		Before	After	Before	After	Before	After
Parasite	Taenia solium	3.494	0.078	1	1	2183	75
	Echinococcus granulosus	0.035	0.003	1	7.7	215	81
	Trichinella zimbabwensis	0.007	0.009	4.71	9.1	17	32
Virus	Human herpesvirus 4	22.233	1.4	1	1	84	3
	Human herpesvirus 4 type 2	20.645	0.9	1	1	32	2

### Sample collection and processing

CSF was collected via standard procedures. DNA was extracted from 300 μL CSF samples using the TIANamp Micro DNA Kit (DP316, Tiangen Biotech, Beijing, China) and sonicated to a size of 200–300 bps fragments (Bioruptor Pico protocols). DNA libraries were then constructed via end-repaired adaptation added overnight and application of polymerase chain reaction (PCR) amplification to extracted DNA. An Agilent 2100 Bio-analyzer (Agilent Technologies, Santa Clara, CA) in combination with quantitative PCR was utilized to quantify DNA libraries. Sequencing was performed using the BGISEQ-100 platform (BGI-Tianjin, Tianjin, China) [5].

### Data analysis

Most high-quality sequencing data were generated and computational subtraction of human host sequences was performed using a Burrows- Wheeler Alignment tool [6]. Remaining sequencing data were aligned with Microbial Genome Databases, which is composed of 2700 whole genome sequences of viral taxa, 1494 bacterial genomes or scaffolds, 73 fungi and 47 parasites associated with human infectivity. The total number of reads from different samples was standardized as 20 M for comparison. After alignment, previously obtained files were filtered and duplicates removed. ThMmapped data were further processed and with the depth and coverage of each species calculated using Soap Coverage (http://soap.genomics.org.cn). A control sample from a non-infected patient was obtained and subjected to the aforementioned procedures. The DNA of CSF samples before and after drug treatment was extracted to construct a complementary DNA (cDNA) library for sequencing. The number of reads from the cDNA library of the patient’s CSF was 37,781,754 (before drug treatment) and 19,043,412 (after drug treatment) respectively. Those of the control sample were 25,962,600. As a result of pathogen detection, *T.solium* was identified as the most predominant parasite with 3.495% coverage of the *T.solium* genome in the first detection; however this reduced to 0.078% in the second detection after specific drug therapy. No reads of *T.solium* were detected from control samples. Reads of viruses were also detected in the CSF samples, later identified as human herpesvirus 4 (HHV-4). After drug therapy, the number of HHV-4 reads decreased approximately 20-fold. The results of CSF NGS prior to and after praziquantel therapy are shown in Table 2.

## Discussion

To our knowledge, this is the first reported case in which NGS was used to detect *T. solium* infection in a patient with neurocysticercosis (NCC) who presented with several months of headache and paroxysmal left face and arm numbness.

Initially, extensive investigations using brain MRI and cerebral MRA did not reveal the cause of her headache. CSF cytological and biochemical examination results as well as increased intracranial pressure readings suggested inflammatory changes; thus viral encephalitis was considered as a probable etiology. However, symptoms did not obviously resolve after antiviral therapy. As the patient’s headache aggravated, she developed a pure sensory stroke due to infarction of the right thalamus. Although cerebral MRV revealed right lateral sinus dysplasia, cerebral venous sinus thrombosis was not considered due to the ineffectiveness of anticoagulant therapy. Her following DSA suggested unremarkable findings. Four subsequent lumbar punctures revealed significant intracranial hypertension and a slightly inflammatory CSF profile with elevated white cells and protein levels. Particularly considering the multiple small, ring-enhanced lesions and meningeal enhancement apparent on brain MRI, we focused our attention to the detection of etiologic microorganisms. Routine tests and cultures for bacteria, mycobacteria (including *Mycobacillus Tuberclosis*) and cryptococcus, however, were negative. The CSF NGS revealed several possible pathogens including HHV-4. Also known as *Epstein-Barr virus* (EBV), HHV-4 is one of the most common human viruses. EBV is able to persist in B lymphocytes of the host for life, but in the vast majority of healthy carriers the virus generally causes no disease [7]. As for the *Trichinella zimbabwensis* and *Echinococcus granulosus*, their coverage were very low, and *T. zimbabwensis* was scarcely reported of causing disease in China. The further CSF parasite antibodies detection confirmed cysticercal antibody positivity but not for the echinococcosis antibody, thus *T. zimbabwensis* and *E. granulosus* were not considered as the pathogen in our case. NCC was then finally diagnosed.

NCC is a common parasitic disease of the CNS caused by the encysted larval stage of the tapeworm *T. solium*. The incidence of cerebrovascular complications in the setting of this pathology was reported to vary 4%~ 12%, with ischemic stroke being the commonest [3]. Cerebrovascular complications of NCC have been found to be closely related to cysticercal distribution and the severity of accompanying arteritis [8]. Considering her normal angiographic findings, this patient could be classified as a case of diffuse NCC with small vessel involvement [3, 8]. Arteritis with progressive stenosis induced by degenerating parasites and chronic subarachnoid inflammation was considered as the main pathologic mechanism [3]. Involved vessels reveal thickening of the adventitia with fibrosis of the media, as well as endothelial hyperplasia, producing subsequent brain infarction [9].

In clinical practice, histological confirmation is not possible in most NCC cases, and diagnosis is usually based on neuroimaging and/or immunological tests of specific anti-*T. solium* antibodies [1]. Despite the availability of modern neuroimaging methods and reliable immunodiagnostic tests, diagnosis of NCC remains challenging, mainly due to variability in sensitivity and specificity of immunodiagnostic tests and a lack of typical neuroimaging findings in the majority of NCC cases. Detection of *T. solium* DNA in CSF by conventional PCR has been reported to have high sensitivity and specificity in Latin America [10, 11], especially, for extraparenchymal NCC [12]. However, such assays typically require an a priori suspicion of *T. solium* infection.

NGS is an unbiased and rapid tool capable of detecting a broad range of bacterial, viral, fungal or parasite DNA sequences simultaneously in clinical samples. We demonstrate here, for the first time, that NGS provided key clues and greatly assisted in accurately diagnosing this patient with NCC. Several studies have reported *T. solium* DNA to persist in CSF for many years, with its DNA load either increasing or decreasing after treatment [11]. Such diverse results may be related to detection time-points, therapeutic strategies and the location of the organism. In our patient, CSF cysticercal antibodies remained positive while tapeworm presence dramatically decreased, by almost 100 fold, on NGS. As her symptoms were effectively relieved, we considered our antiparasitic treatment to have been effective. As reported, antibody-detecting techniques do not have capacity to distinguish between exposed, inactive or active infection, and exhibit a low positive predictive value in cases with viable cysticercosis [13]. Therefore, genetic analysis via NGS should be considered by clinicians to potentially be a more sensitive diagnostic tool. Further studies are needed to determine if CSF DNA load can be used to evaluate the effectiveness of treatment and optimal timing of therapy.

## Conclusions

This study indicated that NGS was a promising tool for the early and accurate diagnosis of central nervous system (CNS) infection, especially when the clinical manifestations are atypical. Further studies are required in order to evaluate the persistence of DNA in the CSF of patients.

## Abbreviations

CNS: Central nervous system
CSF: Cerebrospinal fluid
DSA: Digital subtraction angiography
EBV: Epstein-Barr virus
HHV-4: Human herpesvirus 4
MRA: Magnetic resonance angiography
MRI: Magnetic resonance imaging
MRV: Magnetic resonance venography
NCC: Neurocysticercosis
NGS: Next Generation Sequencing
PCR: Polymerase chain reaction
